# Ethyl pyruvate inhibits glioblastoma cells migration and invasion through modulation of NF-κB and ERK-mediated EMT

**DOI:** 10.7717/peerj.9559

**Published:** 2020-07-21

**Authors:** Qing Huang, Yongming Fu, Shan Zhang, Youxiang Zhang, Simin Chen, Zuping Zhang

**Affiliations:** 1 Department of Pathogen Biology, School of Basic Medicine, Central South University, Changsha, China; 2 Department of Infectious Diseases and Hunan Key Laboratory of Viral Hepatitis, Xiangya Hospital, Central South University, Changsha, China

**Keywords:** Ethyl pyruvate, Migration, Invasion, EMT (epithelial–mesenchymal transition), NF-κB, ERK, Glioblastoma

## Abstract

**Background:**

Glioblastoma is a grade IV glioma with the highest degree of malignancy and extremely high incidence. Because of the poor therapeutic effect of surgery and radiochemotherapy, glioblastoma has a high recurrence rate and lethality, and is one of the most challenging tumors in the field of oncology. Ethyl pyruvate (EP), a stable lipophilic pyruvic acid derivative, has anti-inflammatory, antioxidant, immunomodulatory and other cellular protective effects. It has been reported that EP has potent anti-tumor effects on many types of tumors, including pancreatic cancer, prostate cancer, liver cancer, gastric cancer. However, whether EP has anti-tumor effect on glioblastoma or not is still unclear.

**Methods:**

Glioblastoma U87 and U251 cells were treated with different concentrations of EP for 24 h or 48 h. CCK8 assay and Colony-Formation assay were performed to test the viability and proliferation. Wound-healing assay and Transwell assay were carried out to measure cell invasion and migration. Western blot was not only used to detect the protein expression of epithelial-mesenchymal transition (EMT)-related molecules, but also to detect the expression and activation levels of NF-κB (p65) and Extracellular Signal Regulated Kinase (ERK).

**Results:**

In glioblastoma U87 and U251 cells treated with EP, the viability, proliferation, migration, invasion abilities were inhibited in a dose-dependent manner. EP inhibited EMT and the activation of NF-κB (p65) and ERK. With NF-κB (p65) and ERK activated, EMT, migration and invasion of U87 and U251 cells were promoted. However the activation of NF-κB (p65) and ERK were decreased, EMT, migration and invasion abilities were inhibited in U87 and U251 cells treated with EP.

**Conclusion:**

EP inhibits glioblastoma cells migration and invasion by blocking NF-κB and ERK-mediated EMT.

## Introduction

Glioblastoma (GBM), a grade IV glioma, originates from neuroglial stem or progenitor cells. It is the most common and fatal primary tumor of the central nervous system, both in adults and children. As a brain tumor, it’s highly malignant with poor prognosis, and extremely high incidence. Glioblastoma is one of the most challenging tumors (Jemal et al., 2010). Because glioblastoma is highly heterogeneous and invasive, it often invades the surrounding normal brain tissue and has no obvious boundary. Owing to that, traditional surgical treatment cannot completely resect the tumor; the radiotherapy and chemotherapy after the procedure is essential. Studies have shown that postoperative chemotherapy can prolong or improve the survival duration and survival rate of patients (Balca-Silva et al., 2015; Qian & Zhu, 2018; Sun et al., 2015b). However, due to the blood brain barrier, many drugs reach the tumor site at a low concentration, which makes it difficult to kill tumor cells (Da Ros et al., 2018). In addition, cell resistance greatly reduces the effectiveness of these commonly used chemotherapy drugs (Da Ros et al., 2018). Therefore, it is necessary to find new effective chemotherapy drugs for glioblastoma.

Ethyl pyruvate (EP), a stable lipophilic pyruvic acid derivative, has anti-inflammatory, antioxidant, immunomodulatory and other cellular protective effects (Chen et al., 2017; Fink, 2007), and has been classified as a harmless substance to human body by Food and Drug Administration (FDA). As an extremely important organic synthesis intermediate, EP is safe, stable and easy to be absorbed by cells (Wang et al., 2012b). It has been widely applied in medicine, food (Bozkurt et al., 2016), cosmetics and other industries. Besides, it has been proved that EP has obvious anti-tumor effects on many types of tumor, including pancreatic cancer, prostate cancer, liver cancer, gastric cancer, melanoma, etc. (Cheng et al., 2014; Huang et al., 2018; Zhang et al., 2016; Zhou & Sakamoto, 2019). The underlying mechanisms of its anti-tumor effects mainly include inhibiting cell proliferation, inducing cell apoptosis, blocking cell cycle, attenuating tumor invasion and migration, suppressing angiogenesis, affecting tumor cell autophagy, etc. (Li et al., 2012; Park et al., 2011; Wang et al., 2012a; Wang et al., 2013; Zhang et al., 2016). However, the underlying effect of EP on glioblastoma cells is yet to be studied.

In the present study, we explored the effect of EP on glioblastoma cells and its potential molecular mechanism.

## Materials and Methods

### Chemicals, reagents, antibodies and cell culture

Ethyl pyruvate, TNF-α and U0126 were purchased from Sigma-adrich, peprotech and Cell Signaling Technology, respectively. Antibodies of NF-κB (p65), Extracellular Signal Regulated Kinase (ERK), E-cadherin, Snail, Twist1, ZEB1, Vimentin were obtained from Wanlei biology (ShenYang, China) and were used as primary antibodies in the Western blot. Transwell was bought from CWBIO (JiangSu, China); Matrigel was purchased from BD Biosciences (San Jose, CA, USA); Human U87 and U251 cell lines were obtained from American type culture collection (Manassas, VA, USA). Cells were maintained in Dulbecco’s Modified Eagle’s Medium (DMEM, Sigma–Aldrich, St. Louis, MO, USA) supplemented with 10% fetal bovine serum (FBS, Gibco, Rockville, MD, USA).

### Cell counting kit-8 assay

Cell viability was evaluated by Cell Counting-Kit 8 (CCK-8) assay. Cells treated with 0, 5, 10, 20 and 40 mM EP were plated into the 96-well plate with 3 × 10^3^ cells per well. After incubation for 48 h, 10 μL CCK-8 solution was added to each well, and the plates were incubated for additional 1 h, the absorbance (A) at 450 nm was detected with a Microplate Reader (Bio-Rad, Hercules, CA, USA).

### Colony formation assay

To determine the effect of EP on glioblastoma cells proliferation, the colony formation assay was conducted. Cells were seeded at a density of 1,500 cells per well and left overnight. The culture medium was then replaced with DMEM containing 0, 10, 20, 30 mM EP and renewed it every 2 or 3 days. A total of 2 weeks later, the colonies formed were subjected to 15 min fixation in 4% paraformaldehyde and 30-min staining in 0.1% violet crystal. After removing the staining solution, the colonies were air-dried, 1mL 1% sodium dodecyl sulfate (SDS) was used to dissolve stained colonies, absorbance of 100 µL SDS solution was measured at 450 nm by a Microplate Reader (Bio-Rad, Hercules, CA, USA).

### Wound-healing assay

To assess cell migration of U251 and U87 cells treated with EP, cells were evenly planted in a 6-well plate with 1 × 10^6^ cells per well and cultured overnight in a cell incubator (37 °C, 5% CO_2_). When the cells covered 90% of the plate bottom area, the plate was vertically scratched with a 10 µL sterile gun head (pay attention to uniform force). After that, the culture medium in the plate was discarded, gently washed with PBS for three times, and the cell debris residue was rinsed off to make sure the visual field clear during photographing. The culture medium containing 1% FBS was added to the six-well plate, and the cells were treated with different concentrations of EP. The healing process of the cell scratch wound was recorded with an inverted microscope. They were photographed 0 h, 12 h and 24 h after EP’s treatment, respectively.

### Transwell migration and matrigel invasion assays

To assess cell migration, 4 × 10^4^ cells in 150 μL DMEM with 1% FBS and indicated concentrations of EP (0, 10, 20 and 30 mM) were seeded in the upper chamber of transwell migration chambers (8 μm BioCoat Control Inserts, Becton Dickinson Labware, Bedford, MA, USA). A total of 600 μL DMEM containing 10% FBS was added into the lower chamber. After 24 h, the cells in the upper chamber were fixed by 4% paraformaldehyde for 20 min at room temperature, stained with 1% crystal violet for 15 min, and photographed in three independent 20× fields for each well. Transwell invasion assay was similarly performed with transwell upper chamber pre-coated with Matrigel. All experiments were performed in triplicate.

### Western blot analysis

Cells were lysed with RIPA lysis buffer (Cell Signaling Technology, Danvers, MA, USA) to extract proteins from U251 and U87 cells. And the concentration of these proteins was quantified with BCA Protein Assay Kit (Beyotime, Shanghai, China). The equal amount of proteins was separated by 10% sodium dodecyl sulfate-polyacrylamide gel electrophoresis (SDS-PAGE) gel and transferred to polyvinylidene difluoride membranes (EMD Millipore, Billerica, MA, USA). Then the membranes were blocked with 5% skim milk for 2 h at room temperature and incubated with primary antibodies (anti-ERK, anti-NF-κB (p65), anti-α-Tublin, anti-ZEB1, anti-E-cadherin, anti-Snail, anti-Twist1 or anti-Vimentin) at 4 °C overnight, followed by incubation with horseradish peroxidase-conjugated secondary antibody at room temperature for 1 h. The proteins bands were visualized by an enhanced chemiluminescence detection kit (Amersham, GE Healthcare, Chicago, IL, USA). Image J image analysis software was applied to calculate the grayscale value of the bands on the images, which were used for statistical analysis.

### Statistical analysis

Statistical analysis was performed using the Graphpad prism8. The measurement data were expressed as mean ± standard deviation (SD). The differences in the groups were analyzed by Ordinary one-way ANOVA Multiple comparisons. *P* < 0.05 was considered statistically significant.

## Results

### EP inhibits U251 and U87 cells viability and proliferation

**Figure 1:**
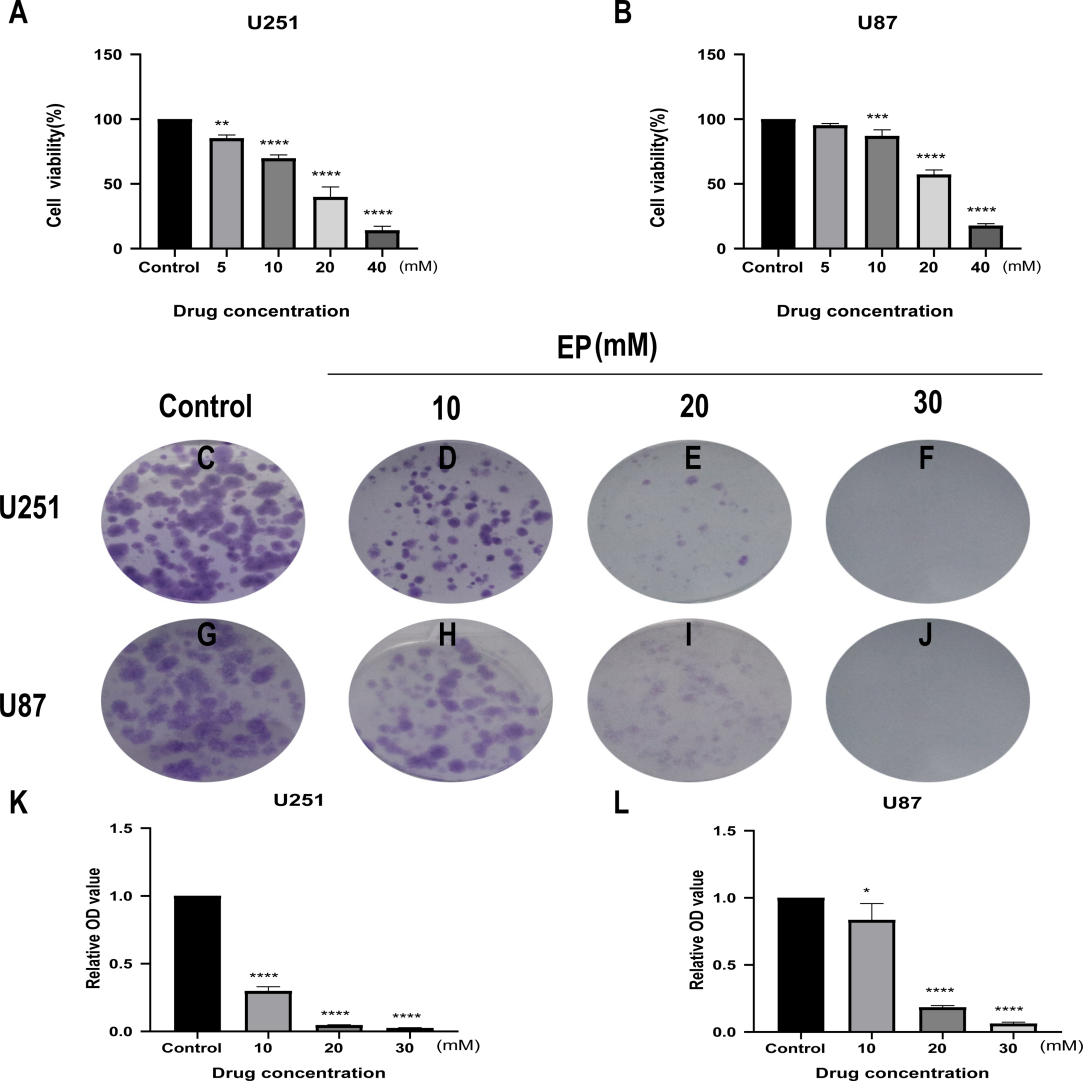
EP inhibits cell viability and colony formation in U251 and U87 cells. (A and B) Effect of EP on cell viability in U251 and U87 cells. CCK8 assay was carried out to detect the cell viability 48 h after cells were treated with increasing doses of EP. EP significantly decreased cell viability in a dose dependent manner. (C–L) Effect of EP on colony formation in U251 and U87 cells. EP significantly decreased the number of colonies in a dose-dependent manner. Cell clones were stained with crystal violet, then the stained clones were dissolved with 1% SDS and the absorbance of the solution at 450 nm wavelength was detected after 10–14 days. Data are represented as mean ± SD of three independent experiments. *p ≤ 0.05, **p ≤ 0.01, ***p ≤ 0.001, ****p ≤ 0.0001 (Ordinary one-way ANOVA Multiple comparisons).

CCK8 assay was performed in U251 and U87 cells to determine the effects of EP on human glioblastoma cell viability. Glioblastoma cells treated with different concentrations of EP **(**0, 5, 10, 20 and 40 mM) for 48 h showed the inhibition of cell viability in a dose-dependent manner (Figs. 1A and 1B).

Next, to detect the effects of EP on the proliferation of U251 and U87 cells, colony formation assays were carried out. For both U251 and U87 cells, absorbance of SDS solution for dissolving cell clones was gradually decreased with the increase of EP concentration (Figs. 1C–1L). These results indicated that EP inhibits U251 and U87 cells viability and proliferation in a dose-dependent manner.

### EP attenuates the migration and invasion of U251 and U87 cells

**Figure 2:**
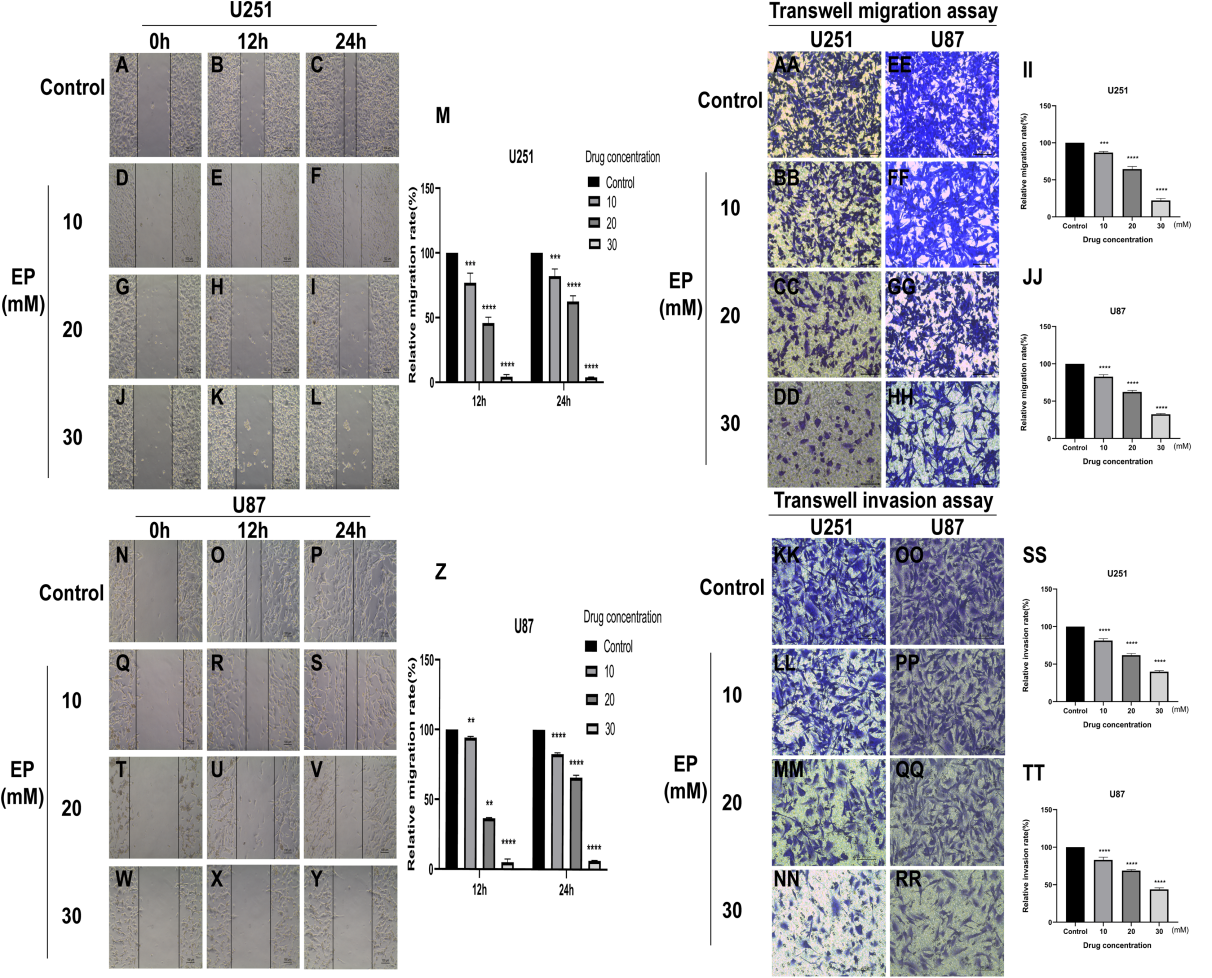
EP attenuate the migration and invasion of U251 and U87 cells. (A–Z) Wound healing assay of U251 and U87 cells after treatment with 0, 10, 20, 30 mM EP for 0, 12 and 24 h. (AA–JJ) Transwell migration assay of U251 and U87 cells after treatment with 0, 10, 20, 30 mM EP for 24 h. (KK–TT) Transwell invasion assay of U251 and U87 cells after treatment with 0, 10, 20, 30 mM EP for 24 h. Data are represented as mean ± SD of three independent experiments. **p ≤ 0.01, ***p ≤ 0.001, ****p ≤ 0.0001 (Two-way ANOVA Multiple comparisons).

To identify whether EP can affect the movement, migration and invasion ability of U251 and U87 cells, scratch assay and transwell assay were conducted. The results of wound healing assay suggested that the migration distance of cells treated with EP is significantly shorter than that of the control and the difference in migration distance increased with the increase of EP concentration. It suggested that EP may affect the migration ability of U251 and U87 cells (Figs. 2A–2Z). Similarly, the number of migrated and invaded cells decreased in U251 and U87 cells treated with different concentrations of EP (Figs. 2AA and 2TT). Those demonstrated EP attenuated migration and invasion abilities of glioblastoma cells.

### EP inhibits EMT of U251 and U87 cells

**Figure 3:**
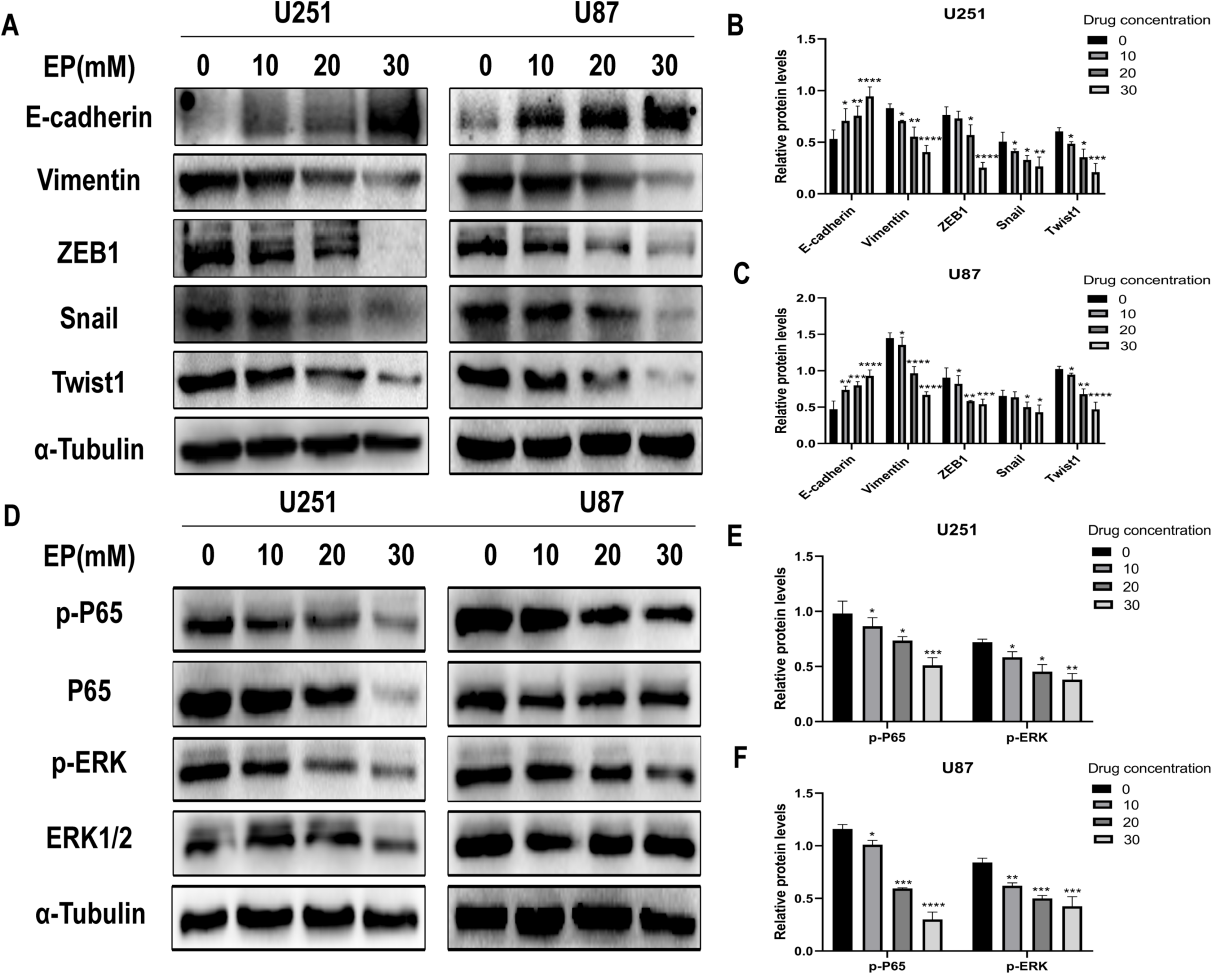
EP inhibits epithelial-mesenchymal transition and inhibits NF-κB (p65) and ERK phosphorylation in U251 and U87 cells. (A–C) EP promotes epithelial marker E-cadherin expression and inhibits the expression of mesenchymal marker Vimentin and EMT related transcription factor ZEB1, Snail, Twist1 in U251 and U87 cells with a dose-dependent manner. All protein levels were evaluated in EP treated cells after 48 h. (D–F) EP inhibits the expression and activation of NF-κB (p65) and ERK in U251 and U87 cells with a dose-dependent manner. All protein levels were evaluated in EP treated cells after 48 h. Data are represented as mean ± SD of three independent experiments. *p ≤ 0.05, **p ≤ 0.01, ***p ≤ 0.001, ****p ≤ 0.0001 (Two-way ANOVA Multiple comparisons).

Considering that migration and invasion is closely related with Epithelial-mesenchymal transition (EMT) of cancer cells, the protein expression of molecules related to this process in U251 and U87 cells with and without EP treatment were detected. After 48 h incubation with EP for the indicated concentrations (0, 10, 20 and 30 mM), the expression of E-cadherin, Vimentin and EMT-related transcription factors ZEB1, Snail and Twist1 were examined. Western blot showed that EP increased the expression of epithelial marker E-cadherin and decreased the levels of mesenchymal associated genes Vimentin, transcription factor ZEB1, Snail and Twist1 (Figs. 3A–3C). These results strongly indicated that EP could apparently promote epithelial properties and remarkably inhibit mesenchymal properties of glioblastoma cells in a dose-dependent manner.

### EP inhibits the activation of NF-κB (p65) and ERK in U251 and U87 cells

In view of abnormally activated ERK (p-ERK) promotes the proliferation, invasion and migration of numerous types of tumor cells (Yuan et al., 2009; Zhang et al., 2016, 2020; Zhou & Sakamoto, 2019), and NF-κB (p65) is a transcription factor closely related to EMT and its activation can significantly promote tumor invasion and angiogenesis. Most importantly, EP has been reported as an inhibitor of NF-κB (Han et al., 2005). Therefore, we speculate whether the mechanism of EP inhibiting EMT in glioblastoma is related to NF-κB (p65) and ERK signaling pathways. The expression level of NF-κB (p65), ERK and their phosphorylated proteins after 48 h treatment with different concentration of EP were measured by Western blot. Results showed that with the increase of EP concentration, the phosphorylation levels of NF-κB (p65) and ERK decreased (Figs. 3D–3F). These results illustrated that EP inhibits the activation of NF-κB (p65) and ERK in glioblastoma cells.

### EP antagonizes NF-κB and ERK-induced EMT of glioblastoma cells

**Figure 4:**
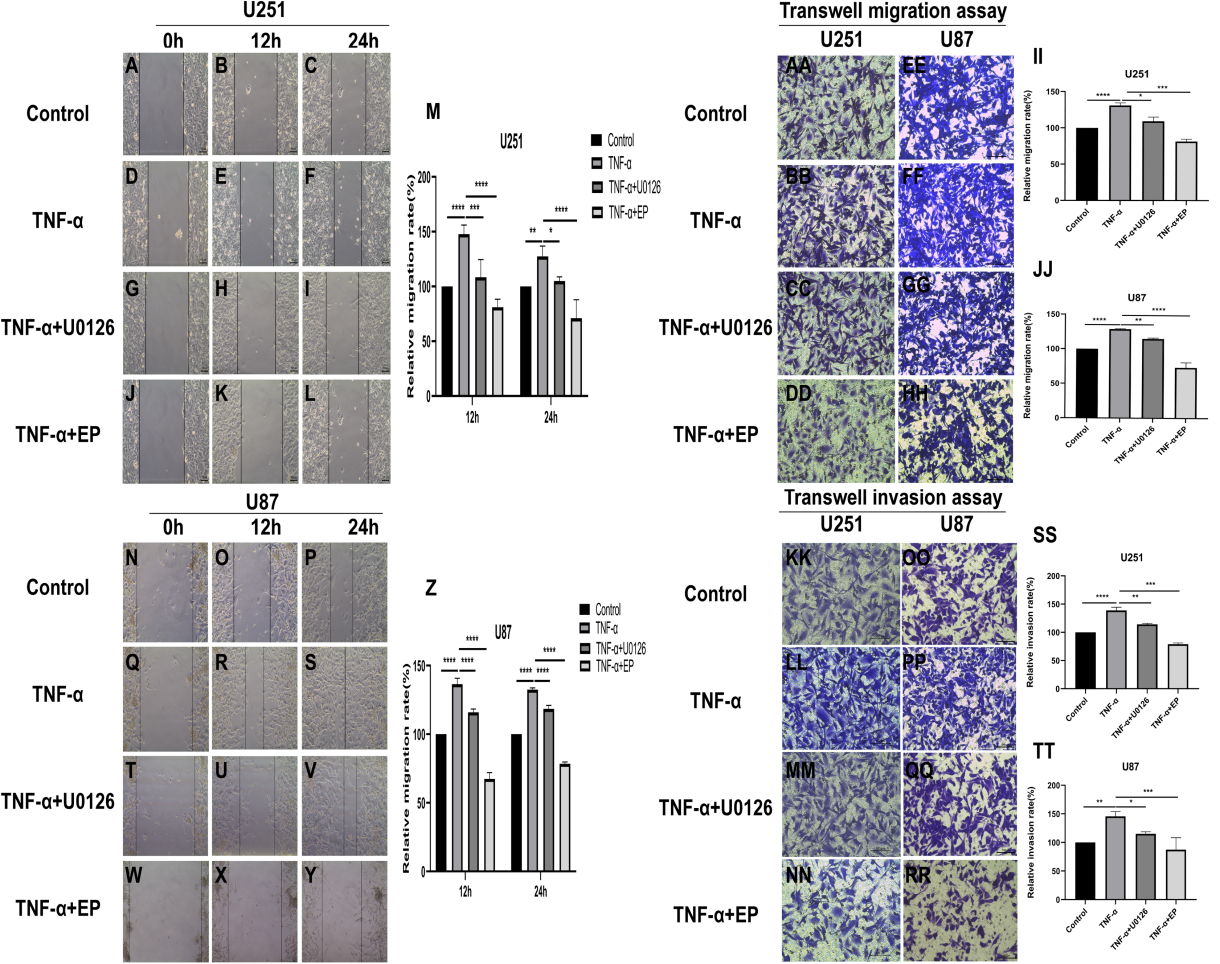
Effects of NF-κB and ERK on U251 and U87 cells migration and invasion ability. (A–Z) Wound healing assay of U251 and U87 cells after treated with control group, TNF-α (10 μM) group, TNF-α coupled with U0126 (10 pg/L) group and TNF-α coupled with EP (20 mM) group for 0, 12 and 24 h. (AA–TT) Transwell migration assay and Transwell invasion assay of U251 and U87 cells after treated with control group, TNF-α (10 μM) group, TNF-α coupled with U0126 (10 pg/L) group and TNF-α coupled with EP (20 mM) group for 24 h. Data are represented as mean ± SD of three independent experiments. *p ≤ 0.05, **p ≤ 0.01, ***p ≤ 0.001, ****p ≤ 0.0001 (Two-way ANOVA Multiple comparisons).

TNF-α is an inflammatory factor that can activate NF-κB and ERK pathways (Ahmad et al., 2019; Ai et al., 2016; Bigatto et al., 2015; Jiang et al., 2015). U0126 is a commonly used ERK inhibitor. To further validate the role of NF-κB (p65) and ERK in EP-inhibited glioblastoma cells EMT, the migration and invasion ability of U251 and U87 cells were tested under four different treatment conditions, including control group (untreated group), TNF-α (10 µM) treatment group, TNF-α coupled with U0126 (10 pg/µL) treatment group, TNF-α coupled with EP (20 mM) treatment group (Figs. 4A–4TT). The results of wound healing assay and transwell assay showed that, compared with the control group, the cell migration and invasion ability in the TNF-α treatment group were significantly increased, while the TNF-α coupled with U0126 treatment group slightly restored this change, and EP further offset the change caused by TNF-α. The above results preliminarily suggested that the activation of NF-κB (p65) and ERK pathways promoted the invasion and migration ability of glioblastoma cells, while EP inhibited the activation of NF-κB (p65) and ERK and further inhibited the invasion and migration of glioblastoma cells.

**Figure 5:**
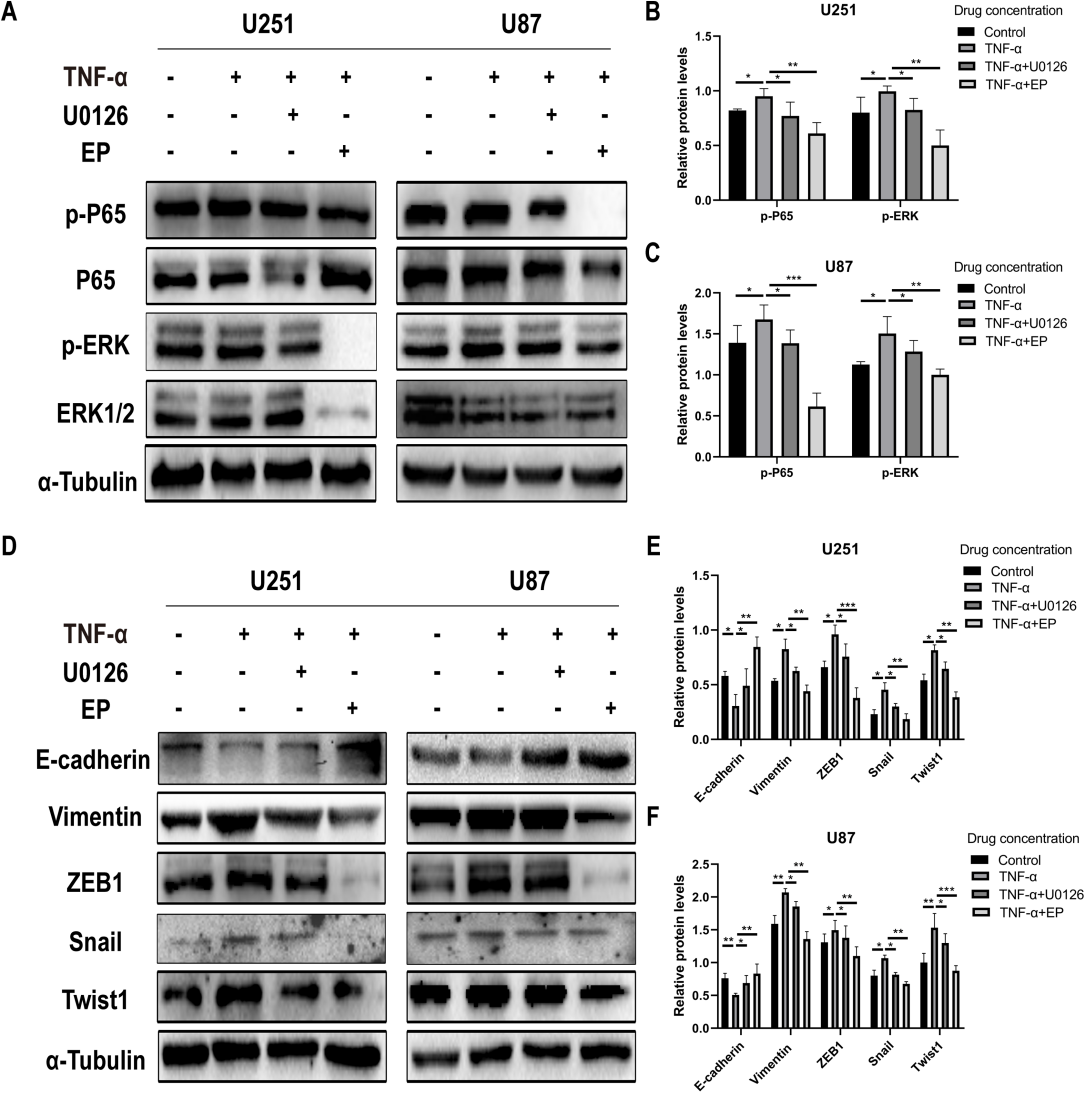
EP inhibitis EMT induced by NF-κB and ERK pathways in U251 and U87 cells. (A–C) The expression level of NF-κB (p65), ERK and their phosphorylated proteins in U251 and U87 cells treated with different conditions including control group, TNF-α (10 μM) group, TNF-α coupled with U0126 (10 pg/L) group, TNF-α coupled with EP (20 mM) group. (D–F) The protein expression levels of E-cadherin and mesenchymal associated genes/activators in U251 and U87 cells treated with different conditions including control group, TNF-α (10 μM) group, TNF-α coupled with U0126 (10 pg/L) group, TNF-α coupled with EP (20 mM) group. Data are represented as mean ± SD of three independent experiments. *p ≤ 0.05, **p ≤ 0.01, ***p ≤ 0.001 (Two-way ANOVA Multiple comparisons).

In order to further explore whether EP inhibits EMT induced by NF-κB and ERK pathways, we not only examined the protein expression levels of E-cadherin and mesenchymal associated genes and transcription factors, but also detected the expression level of NF-κB (p65), ERK and their phosphorylated proteins in U251 and U87 cells in control group, TNF-α treatment group, TNF-α coupled with U0126 treatment group and TNF-α coupled with EP (20 mM) treatment group. The results showed that when NF-κB (p65) and ERK were activated, E-cadherin was significantly down-regulated, while ZEB1, Vimentin, Snail and Twist1 were significantly up-regulated. When U0126 was added, ERK phosphorylation was inhibited, the down-regulation degree of E-cadherin and the up-regulation degree of ZEB1, Vimentin, Snail and Twist1 were weakened. When cells were treated with EP instead of U0126, the activation of NF-κB (p65) and ERK was inhibited. Compared with the experimental group treated with only TNF-α, EP significantly increased the expression of E-cadherin and significantly decreased the expression of ZEB1, Vimentin, Snail and Twist1 (Figs. 5A–5F). The above results identified that EP inhibited EMT induced by NF-κB and ERK pathways in U251 and U87 cells.

## Discussion

Glioblastoma is classified by WHO as a grade IV glioma with the highest degree of malignancy due to its highly invasive and growth mode. Glioblastoma is the most common and fatal primary tumor of the central nervous system, with an incidence rate of about 3/100,000 and a median survival period of less than 15 months (Wesseling & Capper, 2018). Although a great deal of studies has been done on the pathogenesis and treatment of glioblastoma in the past few decades, the efficacy of clinical therapies is still not optimistic. At present, the main treatment for glioblastoma is the combination of surgery and radiochemotherapy. Because the lesion often cannot be completely removed by surgery, subsequent radiotherapy and chemotherapy are very important for killing residual tumor cells and prolonging the survival time of patients (Binabaj et al., 2018; Sun et al., 2015b). The commonly used chemotherapeutic drugs for clinical treatment of glioblastoma are temozolomide, nimustine, bevacizumab, etc. However, due to the blood-brain barrier, drug toxicity, drug resistance and some other problems (Felsberg et al., 2009; Happold et al., 2012; Taylor, Brzozowski & Skelding, 2019), the effectiveness of traditional drugs is more or less limited. Therefore, it is of great significance to explore novel chemotherapy drugs for glioblastoma.

Pyruvate is an important glycolytic metabolic intermediate with high scavenging ability of reactive oxygen species (ROS) and hydrogen peroxide. It can improve the oxidative damage of cells and organs induced by ROS, but it is very unstable in aqueous solution. However, as an esterified derivative of pyruvate, EP not only has similar pharmacological activity as pyruvate, but also has better stability. One of the advantages of EP over other compounds is that it is non-toxic and safe for both animal models and humans, which has been confirmed in several previous studies (Bennett-Guerrero et al., 2009). EP has been widely used, not only for cosmetics and food flavor additives, but also as a pharmaceutical intermediate to synthesize antihypertensive drugs, pesticides and some other drugs because of its special bifunctional structure and chemical properties. EP is a small molecule fat-soluble substance. A large number of animal experimental studies have found that intraperitoneal injection of EP can play a neuroprotective role on hippocampal neuron death, spinal cord ischemic injury and dopaminergic cell death by alleviating inflammatory reaction and protecting nerve cells, suggesting that EP can readily penetrate the blood-brain barrier (Choi, Lee & Jeong, 2010; Wang et al., 2009; Wang et al., 2013). Initially, EP attracted much attention for its antioxidant and anti-inflammatory effects, known as the scavenger of reactive oxygen in the body. Inflammatory factors play an important role in the occurrence and development of tumors. As an anti-inflammatory drug and an effective inhibitor of inflammatory mediator late high mobility group box 1 (HMGB1), EP has also been proved to have anti-tumor effects in various tumors in recent years. EP induces apoptosis and cell-cycle arrest in G phase in hepatocellular carcinoma cells by inhibiting the HMGB1–RAGE and AKT pathways (Cheng et al., 2014); EP suppresses growth and invasion of gallbladder cancer cells via downregulation of HMGB1-RAGE axis (Li et al., 2012). Similarly, in the cytoplasm of lung cancer, EP suppressed the growth, invasion and migration and induced apoptosis of NSCLC cells via the HMGB1/RAGE axis and the NF-κB/STAT3 pathway (Liu et al., 2019). EP can also regulate the gastric cancer cell cycle by regulating the P53 signaling pathway (Zhang et al., 2016). However, at present, there are few reports about the effect of EP on glioblastoma and its mechanism. Therefore, we chose two glioblastoma cell lines U251 and U87 to detect the effect of EP on glioblastoma in our present study.

First, in order to detect whether EP affects the biological function of glioblastoma cells, a series of experiments were performed, including CCK8, clone formation assays, wound-healing assay and transwell assay. The results showed that EP could inhibit the viability, proliferation, migration and invasion of glioblastoma cells. The highly invasive nature of glioblastoma is an important reason for its incurability and recurrence, and EMT is closely related to tumor migration and invasion, especially for glioblastoma. So whether EP affects the migration and invasion of glioblastoma by affecting EMT process is deserved to explore.

Epithelial-mesenchymal transition is a reversible biological process characterized by the loss of apical-basal polarity of the epithelial cells and the mesenchymal cells that simultaneously acquire high migration and invasive ability (Salton, Volpe & Confalonieri, 2019). It is a key link in tumor cell invasion and metastasis and is closely related to the occurrence and development of tumors (Kalluri & Weinberg, 2009). Nowadays, a large number of studies have proved that EMT or EMT(-like) plays an important role not only in the in-situ invasion and distant metastasis of breast cancer, cervical cancer, gastric cancer and some other tumors (Jiang et al., 2019; Liu et al., 2017; Nath et al., 2019; Nishizuka, Komada & Imagawa, 2019; Sun et al., 2018; Yilmaz & Christofori, 2009), but also in the invasive growth of malignant glioma (Iser et al., 2017; Mikheeva et al., 2010; Takashima, Kawaguchi & Yamanaka, 2019). In the EMT process, the cell polarity disappears, the adhesion weakens and it morphologically transforms from epithelial cells to mesenchymal cells. At the same time, it is accompanied by changes in various molecular expression levels. For example, the expression of epithelial molecular marker E-cadherin decreased and expression of mesenchymal molecular markers such as N-cadherin and Vimentin were up-regulated. Among these molecules, E-cadherin, a transmembrane glycoprotein, mainly takes part in the process of specific adhesion and connection between intercellular substances, and is a surface marker of EMT in cancer cells (Gheldof & Berx, 2013). Vimentin, a cytoskeletal component of cells, is mainly expressed in mesenchymal derived cells and some undifferentiated cells. It regulates cell migration and adhesion by participating in cytoskeletal recombination during EMT, and is a marker of EMT occurrence (Sun et al., 2015a). In addition, Snail and Twist1 are import transcription factors that regulate EMT. Snail can inhibit the expression of E-cadherin by competitively binding with smad interaction protein to the E-box linking motif at the E-cadherin promoter site. Miyoshi et al. (2004) found that Snail not only inhibits the expression of E-cadherin, but also indirectly regulates the expression of MMPs members to increase the invasion ability of liver cancer cells. Twist is a transcription repressor of the basic helix-loop-helix family, among which twist1 is upregulated in various types of tumors, which can regulate tissue reconstruction and cells migration. Experiments have proved that twist1 can promote the invasion ability of human glioma through epithelial-mesenchymal transformation (Mikheeva et al., 2010). Zinc finger E-box binding homeo box (ZEB) is also one of the important transcription factors related to EMT, including ZEB1 and ZEB2. A recent study on liver cancer showed that ZEB1 could bind to the Vimentin promoter and regulate the transcription of the Vimentin (Qin et al., 2019).

To verify the hypothesis that EP may affect the migration and invasion ability of glioblastoma cells by regulating the EMT process, western blot was used to detect the protein expression levels of EMT-related molecules, including epithelial marker E-cadherin, mesenchymal marker Vimentin and EMT-related transcription factors Snail, Twist1 and ZEB1 in U251 and U87 cells after EP treatment at different concentrations for 48 h.

The results of western blot showed that the protein expression of epithelial marker E-cadherin increased and mesenchymal marker Vimentin, EMT-related transcription factors Snail, Twist1, ZEB1 decreased gradually with the increase of EP concentration. Based on this result, we can speculate that EP inhibits the migration and invasion of glioblastoma cells by inhibiting the occurrence process of EMT, and this inhibition effect was strengthened with the increase of EP concentration.

Nuclear transcription factor NF-kappaB (NF-κB), an important transcription factor, can regulate the expression of various genes related to inflammation (DiDonato, Mercurio & Karin, 2012), anti-apoptosis, angiogenesis (Park et al., 2011), tumor formation and transformation. NF-κB is a heterodimer composed of p50 and p65 subunits. NF-κB activation has a significant promotion effect on tumor metastasis, and it is also a transcription factor closely related to EMT. Min et al. (2008) found that NF-κB can combine with the promoter sequence of vimentin gene to promote the occurrence of EMT by promoting the expression of twist. Chua et al. (2007) found that NF-κB can up-regulate the expression of ZEB1, inhibit the production of a variety of important epithelial differentiation and cell adhesion factors, reduce the expression of E-cadherin, and induce the occurrence of EMT. As an inhibitor of NF-κB, EP can inhibit NF-κB-dependent signaling by directly targeting the subunit p65 of NF-κB (Han et al., 2005). ERK, an important molecule in the multilevel kinases cascade reaction process of MAPK signal transduction pathway, is related to cancer, inflammation, immune diseases and neurodegenerative diseases (Pearson et al., 2001). ERK1/2 is an important serine/threonine protein kinase, whose activity and expression level in various tumor cells such as liver, gastric and cervical cancer are significantly up-regulated (Hwang et al., 2004; Yuan et al., 2009). Erk1/2, mainly activated by mitogens such as various growth factors, can enter the nucleus to act on transcription factors, enhance the transcription and expression of certain genes, promote cell proliferation, differentiation, migration and invasion, and inhibit cell apoptosis (Tanimura & Takeda, 2017). Piette et al. (2009) found that macrophage migration inhibitory factor enhanced the migration and invasion ability of glioma U373 MG cells by activating ERK1/2 signaling pathway. Dexamethasone attenuates the migration and invasion of glioma U373MG cells through GR receptor-mediated inhibition of ERK1/2 pathway. Therefore, we can assume that the mechanism of EP inhibiting EMT of glioblastoma is related to NF-κB and ERK signaling pathways. To verify this hypothesis, we first detected the expression and activation levels of NF-κB (p65) and ERK of glioblastoma U251 and U87 cells treated with different concentrations of EP by western blot. The results showed that the activation of NF-κB (p65) and ERK protein levels gradually decreased in U251 and U87 cells with the increase of EP concentration, which meant that the mechanism of EP inhibiting EMT in glioblastoma may be related to NF-κB and ERK signaling pathways. Then, to further confirm this conjecture, the expression levels of total protein and phosphorylated protein of NF-κB (p65) and ERK in U251 and U87 cells in control group, TNF-α treatment group, TNF-α coupled with U0126 treatment group, TNF-α coupled with EP treatment group were detected. Migration and invasion abilities under these four different conditions were also tested. TNF-α, a classic pro-inflammatory cytokine, is mainly produced in immune cells and participates in inflammation and immune regulation. The pro-inflammatory effect of TNF-α mainly lies in its ability to activate NF-κB in almost all cell types, leading to the expression of inflammatory genes. Previous studies proved that TNF-α and NF-κB can promote the expression of each other (Sokolova & Naumann, 2017). In our study, the results of western blot showed that the levels of phosphorylated NF-κB (p65) and phosphorylated ERK protein were increased in U251 and U87 cells treated with exogenous TNF-α. Correspondingly, the results of scratch assay, transwell, migration and invasion all proved that the migration and invasion ability of glioblastoma U251 and U87 cells were significantly enhanced, which indicated that TNF-α promoted the activation of NF-κB and ERK pathway, and in turn promoted the migration and invasion ability of glioblastoma cells. However, the additional addition of ERK inhibitor U0126 weakened this change because it inhibited the activation of ERK pathway. Interestingly, EP completely reversed the promotion of migration and invasion ability caused by TNF-α, and turned it into the inhibition of migration and invasion ability. Of course, the activation levels of NF-κB (p65) and ERK protein also had corresponding changes. According to these results, we can prove that EP does inhibited the migration and invasion of glioblastoma by regulating NF-κB and ERK pathway.

Finally, to verify whether EP regulates the process of EMT through NF-κB and ERK pathway and thus inhibits the migration and invasion of glioblastoma cells, the protein expression of E-cadherin, Vimentin, Snail, Twist1 and ZEB1 in control group, TNF-α treatment group, TNF-α Coupled with U0126 Treatment Group, TNF-α coupled with EP treatment group was detected. The activation of NF-κB and ERK decreased the expression of E-cadherin but increased that of Vimentin, Snail, Twist1 and ZEB1. U0126 restored the change to a certain extent, while EP completely offset the change and even inhibited the occurrence process of glioblastoma EMT. Therefore, above results suggested at the molecular level that EP attenuates glioblastoma cells migration and invasion ability by inhibiting the NF-κB and ERK-induced EMT.

## Conclusion

In general, our research found that EP has anti-tumor effect in glioblastoma. To be different from the currently reported anti-tumor effect of EP mainly related to HMGB1, our research found that it can inhibit the migration and invasion by weakening NF-κB and ERK-induced EMT in U251 and U87 cells. However, we only tested this in vitro and further evidence in vivo will be explored in our follow-up work.

EP, listed as a safe substance for human body by FDA, has broad prospects for drug development due to its stability and good blood-brain barrier permeability. According to our results, it is expected to become a strategy for clinical treatment of glioblastoma.

## Supplemental Information

10.7717/peerj.9559/supp-1 Supplemental Information 1 The raw data of CCK8.

10.7717/peerj.9559/supp-2 Supplemental Information 2 The raw data of Colony formation assay.

10.7717/peerj.9559/supp-3 Supplemental Information 3 The raw data of western blot.

10.7717/peerj.9559/supp-4 Supplemental Information 4 The raw data of apotosis experiment.

## Additional Information and Declarations

### Data Availability

The following information was supplied regarding data availability: The raw data is available in the Supplemental Files.
