# Supplementary figures and images for: Ethyl pyruvate inhibits glioblastoma cells migration and invasion through modulation of NF-κB and ERK-mediated EMT
Source: PeerJ. 2020 Jul 21;8:e9559. doi: 10.7717/peerj.9559 (PMC7380274; doi:10.7717/peerj.9559)

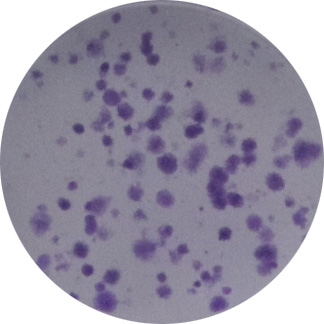

Supplement: Supplemental Information 2 [file peerj-08-9559-s002.zip › Colony formation assay/U251/10-1.jpg]

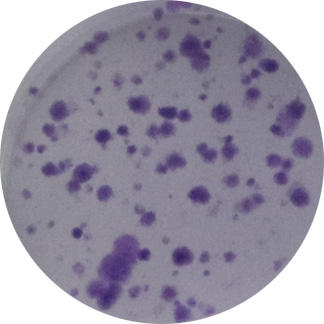

Supplement: Supplemental Information 2 [file peerj-08-9559-s002.zip › Colony formation assay/U251/10-2.jpg]

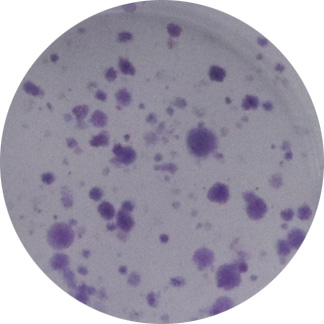

Supplement: Supplemental Information 2 [file peerj-08-9559-s002.zip › Colony formation assay/U251/10-3.jpg]

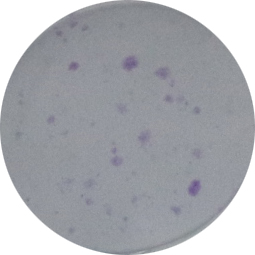

Supplement: Supplemental Information 2 [file peerj-08-9559-s002.zip › Colony formation assay/U251/20-1.jpg]

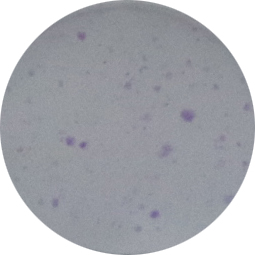

Supplement: Supplemental Information 2 [file peerj-08-9559-s002.zip › Colony formation assay/U251/20-2.jpg]

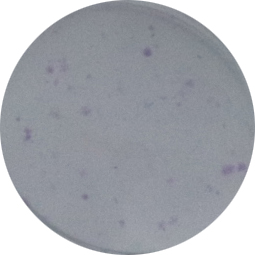

Supplement: Supplemental Information 2 [file peerj-08-9559-s002.zip › Colony formation assay/U251/20-3.jpg]

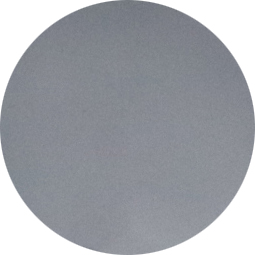

Supplement: Supplemental Information 2 [file peerj-08-9559-s002.zip › Colony formation assay/U251/30-1.jpg]

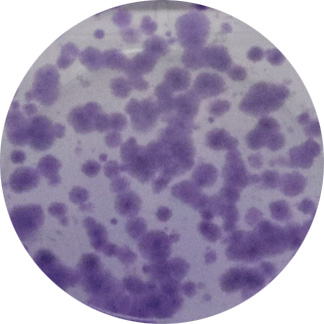

Supplement: Supplemental Information 2 [file peerj-08-9559-s002.zip › Colony formation assay/U251/Control-1.jpg]

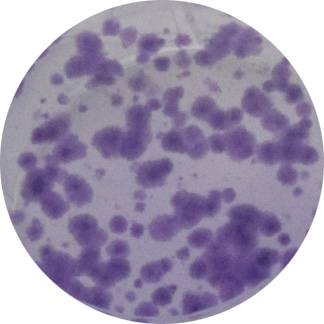

Supplement: Supplemental Information 2 [file peerj-08-9559-s002.zip › Colony formation assay/U251/Control-2.jpg]

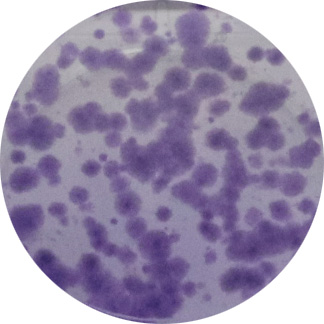

Supplement: Supplemental Information 2 [file peerj-08-9559-s002.zip › Colony formation assay/U251/Control-3.jpg]

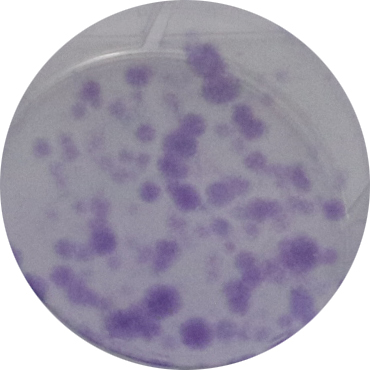

Supplement: Supplemental Information 2 [file peerj-08-9559-s002.zip › Colony formation assay/U87/10-1.jpg]

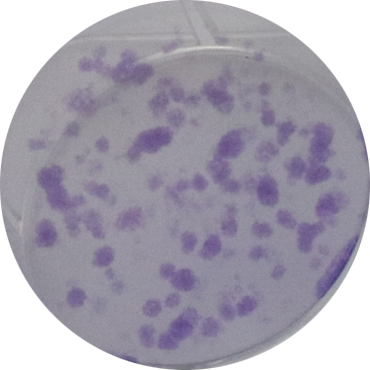

Supplement: Supplemental Information 2 [file peerj-08-9559-s002.zip › Colony formation assay/U87/10-2.jpg]

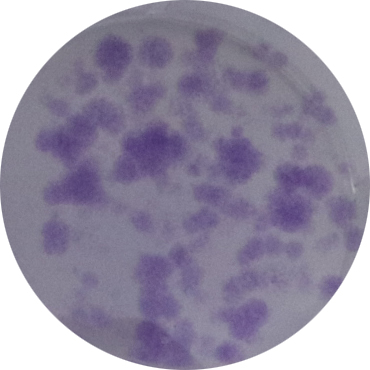

Supplement: Supplemental Information 2 [file peerj-08-9559-s002.zip › Colony formation assay/U87/10-3.jpg]

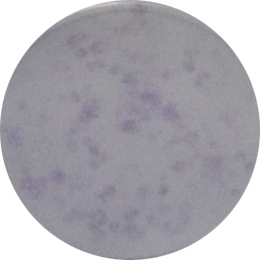

Supplement: Supplemental Information 2 [file peerj-08-9559-s002.zip › Colony formation assay/U87/20-1.jpg]

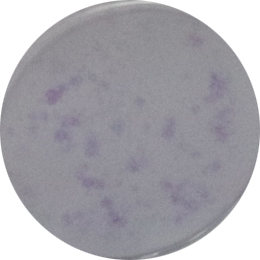

Supplement: Supplemental Information 2 [file peerj-08-9559-s002.zip › Colony formation assay/U87/20-2.jpg]

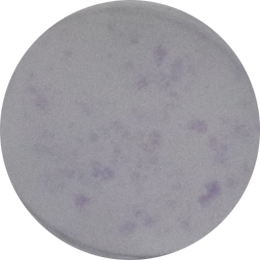

Supplement: Supplemental Information 2 [file peerj-08-9559-s002.zip › Colony formation assay/U87/20-3.jpg]

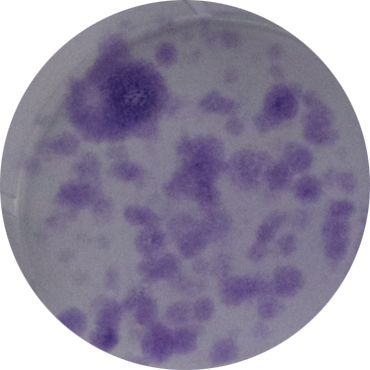

Supplement: Supplemental Information 2 [file peerj-08-9559-s002.zip › Colony formation assay/U87/control-1.jpg]

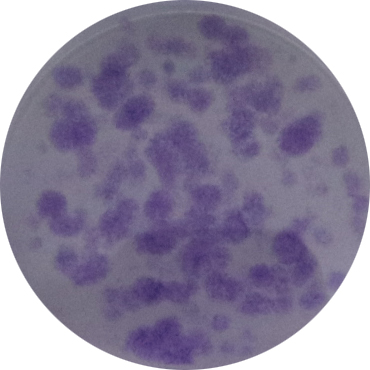

Supplement: Supplemental Information 2 [file peerj-08-9559-s002.zip › Colony formation assay/U87/Control-2.jpg]

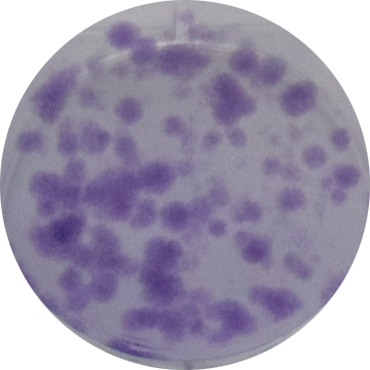

Supplement: Supplemental Information 2 [file peerj-08-9559-s002.zip › Colony formation assay/U87/control-3.jpg]

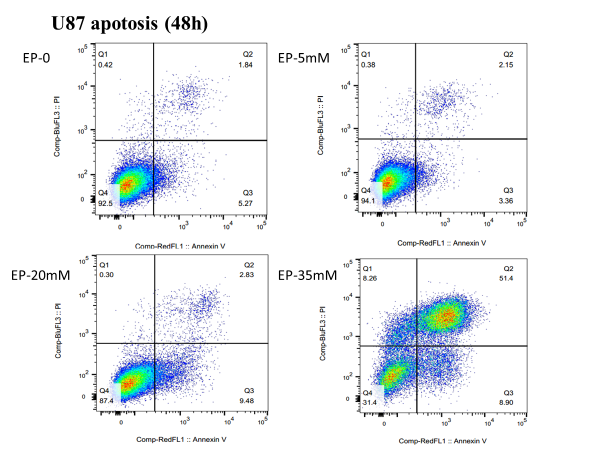


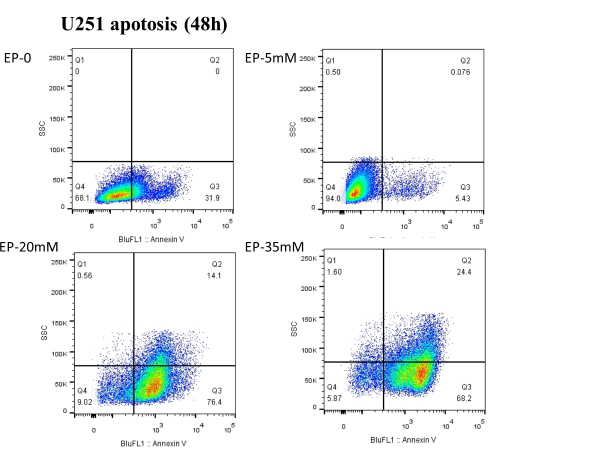

Supplement: Supplemental Information 4 [file peerj-08-9559-s004.docx]
